# Examining cognition in action: laboratory-based attentional control tasks predict performance on combat-relevant tasks in an augmented reality training environment

**DOI:** 10.3389/fpsyg.2025.1543161

**Published:** 2025-05-05

**Authors:** Anthony P. Zanesco, Ekaterina Denkova, Jordan Barry, Costanza Alessio, Amishi P. Jha

**Affiliations:** ^1^Department of Psychology, University of Kentucky, Lexington, KY, United States; ^2^Department of Psychology, University of Miami, Coral Gables, FL, United States; ^3^Department of Psychology, Florida International University, Miami, FL, United States

**Keywords:** working memory, attention, marksmanship, military, augmented reality, human machine interaction

## Abstract

**Introduction:**

There is growing interest in understanding how individual differences in cognitive abilities contribute to military performance. Laboratory-based cognitive tasks, which are well-suited for assessing specific cognitive capacities, offer a controlled and efficient approach for evaluating these differences. If performance on such tasks corresponds with operationally relevant performance, these measures could serve as valuable tools for evaluation, selection, and targeted training interventions to enhance military readiness. Here, we examined associations between performance on laboratory tasks of attentional control and operationally relevant tasks in an augmented reality military training environment.

**Methods:**

Across two study rounds, 45 squads of active-duty U.S. Army soldiers (*N* = 356) completed two laboratory-based tasks of attentional control and a series of operationally relevant drills, requiring attentional control.

**Results:**

Soldiers’ performance on sustained attention and working memory tasks was positively correlated with their performance on operationally relevant drills. Specifically, in both rounds, individuals with greater sustained attention task accuracy performed better on a Shoot/Do Not Shoot drill.

**Conclusion:**

The results indicate that laboratory-based attentional control tasks can serve as useful indicators of performance in military operationally relevant drills. Furthermore, these findings suggest that individual differences in attentional control may influence operationally relevant performance.

## 1 Introduction

For most individuals, lapses in attention are common, yet typically inconsequential in daily life. However, in military operational contexts, such lapses could jeopardize critical mission objectives and result in unnecessary loss of life. To ensure mission success, service members must maintain critical information in mind, identify targets accurately, manage distractions, and focus on task-relevant aspects of the environment. Although cognitive functioning has long been recognized as vital for mission success—playing a key role in the selection of elite forces and military occupational specialties (e.g., Beal, 2010; Picano et al., 2017; Martin et al., 2020)—the specific contributions of attentional control processes remain understudied as predictors of operationally relevant performance in military settings.

Attentional control is a core cognitive capacity that enables the regulation of goal-directed behavior while minimizing the impact of both external and internal distractions, such as environmental stimuli or off-task thoughts (Kane et al., 2007; Oberauer, 2024). This capacity involves both attention and working memory, which are generally acknowledged as interrelated cognitive constructs, although theoretical models differ in how their relationship is characterized (Oberauer, 2019). Individual differences in attentional control, typically measured through tasks of attention and working memory, predict how effectively a person can learn and perform complex tasks in everyday contexts (Cowan, 2014; Draheim et al., 2022). These control functions are also crucial for our ability to engage in many higher-order cognitive functions (Burgoyne and Engle, 2020), such as planning and decision-making (e.g., Bechara and Martin, 2004; Hinson et al., 2003), as well as emotion regulation (e.g., Schmeichel and Tang, 2015).

One benefit of laboratory-based assessment of attentional control is that the level and type of demands can be meaningfully and parametrically manipulated. For example, a single working memory task can be designed to evaluate individual differences in encoding and maintenance processes, as well as distractor inhibition (see Jha et al., 2010). Tasks of attentional control might therefore prove useful for assessing operationally relevant abilities in military service members and refining the parameters considered for military selection. In addition, laboratory-based cognitive task performance could be used to identify training programs that may bolster operationally relevant military performance, without having to collect data from costly and dangerous live-fire field exercises. That is, laboratory-based cognitive tasks could allow for efficient vetting of training programs. These benefits, however, depend on the ability of laboratory-based tasks to assess domain-general aspects of cognition that correspond with the use of those same cognitive functions in real-world situations.

Only a handful of studies have examined this topic, albeit largely in non-military, civilian samples. For example, one research group (Biggs, 2022; Biggs et al., 2015; Biggs and Pettijohn, 2021) has demonstrated positive associations between commission errors on laboratory-based tasks requiring attentional control, involving goal maintenance and response inhibition, and commission errors in simulated shooting scenarios in civilian, adult samples. Similarly, studies in samples of undergraduates and police officers have observed negative associations between working memory capacity and the tendency to incorrectly fire on unarmed targets (Brewer et al., 2016; Kleider and Parrott, 2009; Kleider et al., 2010). In addition, laboratory-based measures of attentional vigilance have been shown to predict performance in computer-based military simulations (Matthews et al., 2013). These findings suggest that laboratory-based measures of attentional control may provide meaningful insights into performance on operationally relevant tasks, highlighting the potential utility of cognitive assessments for military contexts.

Fewer studies have examined associations between laboratory-based measures of attentional control and operationally relevant performance in active-duty military cohorts. One recent study, however, employed elastic net regression to examine a comprehensive set of biological, psychological, and cognitive predictors of military-relevant variables in active-duty service members, including measures of physical aptitude, communication, marksmanship, and operational performance in military field settings (Brunye et al., 2024). Cognitive traits, including aspects of attentional control, such as inhibitory control and working memory, were consistent predictors of operational outcomes.

In the present study, we examined associations between laboratory-based cognitive task performance and operationally relevant performance in a high-fidelity augmented reality military simulation. The study involved two rounds with 45 total squads of enlisted, active-duty U.S. Army infantry soldiers (*N* = 356). Soldiers completed laboratory-based measures of sustained attention and working memory to assess attentional control. In a separate session, their operationally relevant performance was evaluated in an augmented reality simulated environment, known as the Conflict Kinetics “Gunfighter Gym,” which was installed at participating military installations for both studies. The augmented reality simulations provide an interactive experience, modifying the perceptual information in the real-world environment by projecting computer-generated digital content into the environment using video projectors. Soldiers interact with the digital elements using a synthetic rifle. The central aim of the study was to determine whether performance on established laboratory-based tasks of sustained attention and working memory reliably predict performance in this simulated setting.

The study hypothesized that soldiers’ performance on the Sustained Attention to Response Task (SART; Robertson et al., 1997), a continuous Go/No-Go task requiring responses to frequent stimuli and inhibition of responses to infrequent stimuli, would be positively correlated with performance on a simulated Shoot/Do Not Shoot drill. This hypothesis is grounded in the assumption that both tasks rely on shared attentional control processes, particularly the ability to inhibit commission errors in response to infrequent or irrelevant stimuli. Additionally, we predicted that performance on a working memory task with distractors (WMDA) would be associated with accuracy on a simulated working memory shoot drill, as both tasks require the maintenance of a critical target in working memory while minimizing the influence of irrelevant distractions. Finally, participants in Round 1 were asked to rate their experiences with the simulation drills to assess their subjective experience and motivation during participation in the drills, providing further context for evaluating performance in these tasks.

## 2 Methods

Squads were recruited as part of a multi-round longitudinal study investigating the implementation of a mindfulness-based training intervention in the military (ClinicalTrials.gov Identifier: NCT04210076). All data reported in the present study were collected prior to participation in any arm of the intervention. All assessments occurred during participants’ duty day. Per Department of Defense regulations regarding soldier compensation during the duty day, soldiers were not compensated beyond their wages for participation in the project. All participants provided informed consent in compliance with the Institutional Review Board of the University of Miami with oversight from the U.S. Department of Defense Human Research Protections Office.

### 2.1 Participants

#### 2.1.1 Study 1

Fifteen whole squads of enlisted active-duty U.S. Army Soldiers (*N* = 124 total soldiers) were recruited and enrolled in the study from several companies within the same brigade on the same military installation. Squads had 8.3 (*SD* = 0.96, range = 7–10) members on average. Twelve squads were comprised primarily of Infantry, while 1 squad was comprised of Indirect Fire Infantry and 2 squads were comprised of Combat Engineers. One hundred seventeen participants provided demographic information: participants were 22.50 (*SD* = 3.79) years old on average and primarily male (94.9%). Two participants held the rank of E-1, whereas 12 were E-2, 45 were E-3, 35 were E-4, 14 were E-5, 8 were E-6, and 1 did not wish to specify.

#### 2.1.2 Study 2

On a different military installation, 30 whole squads of enlisted active-duty U.S. Army Soldiers (*N* = 232 total soldiers) were recruited and enrolled in the study from several companies within the same brigade. Squads had 7.7 (*SD* = 0.91, range = 7–10) members on average. Squads were comprised of Infantry soldiers. Two hundred and thirty-one participants provided demographic information: participants were 23.32 (*SD* = 4.02) years old on average and primarily male (98.70%). Three participants held the rank of E-1, whereas 21 were E-2, 88 were E-3, 74 were E-4, 34 were E-5, 11 were E-6.

### 2.2 Procedure

During a week-long assessment period in each study, participants completed: (1) either an online laboratory-based assessment (Study 1) or an in-person laboratory-based assessment (Study 2) that included two cognitive tasks and a series of questionnaires that are outside the scope of this project, and (2) a series of in-person operationally relevant drills in an augmented reality simulation environment.

### 2.3 Laboratory-based cognitive tasks

Task assessment sessions were administered via the web-based platform Inquisit,^1^ which took roughly 1.5–2 h to complete. For the online assessment (Study 1), participants received an individualized hyperlink via email at the beginning of the assessment week enabling them to complete the web-based assessment session during the week-long assessment period. For the in-person laboratory-based assessment (Study 2), each squad of participants completed the assessment in a group setting in a quiet classroom on the military installation. Participants were seated approximately 57 cm from their own Apple iPad, and testing was proctored by a team of 1–3 experimenters. The two cognitive tasks are described in detail below.

#### 2.3.1 Sustained Attention to Response Task (SART)

Sustained attention and response inhibition were assessed using a modified version of the Sustained Attention to Response Task (SART; Robertson et al., 1997). During the task, single digits (0 through 9) were continuously presented on screen one at a time for 250 ms. Each digit was followed by a fixation cross that was present for 900 ms during the inter-trial-interval. Participants were instructed to press the spacebar for all digits (Go non-target stimuli) except the number 3 (No-Go target stimuli), while emphasizing both accuracy and speed. Responses were recorded during the stimulus display and the inter-trial interval. Stimuli were presented in black font on a white screen. Trial order was quasi-randomized so that No-Go trials were always separated by at least six other Go trials. After an 80-trial practice block, participants completed two experimental blocks, which consisted of a total of 592 non-targets, 30 targets, and 30 sets of mind wandering probe questions.

Sets of two consecutive mind wandering probe questions were dispersed throughout the task. The first probe (Probe 1) asked, “Where was your attention focused just before the probe?” with participants responding using a 5-point scale ranging from 1 (on task) to 5 (off task). The second probe (Probe 2) asked participants to “characterize what you were thinking about just before this question” by selecting among 7 possible categories. The categories included (1) “I was totally focused on the current task,” (2) “I thought about my performance on the task,” (3) “I was distracted by sights/sounds/physical sensations,” (4) “I had negative thoughts unrelated to the task,” (5) “I had neutral thoughts unrelated to the task,” (6) “I had positive thoughts unrelated to the task,” and (7) “My mind was blank.” The probe questions were displayed until a response was provided.

SART performance was assessed by calculating *A′* (Stanislaw and Todorov, 1999), a nonparametric measure of sensitivity based on a composite of the number of correct hits (correctly withholding a response to No-Go target trials) and false alarms (incorrectly withholding a response to Go non-target trials). Results from the practice block were excluded from all analyses. In Study 1, 110 participants in total were assessed with the SART. Four participants were subsequently removed from SART analyses because they correctly responded to <66% of Go (i.e., non-target) stimuli. Seven additional participants were removed from SART analyses because they had *A′* values <0.5. In Study 2, 231 participants were assessed with the SART. Eleven participants were subsequently removed from SART analyses because more than 40 trials were lost due to a technical error in the administration of the task. One additional participant was removed because they correctly responded to <66% of Go (i.e., non-target) stimuli, and 3 additional participants were removed from SART analyses because they had *A′* values <0.5.

#### 2.3.2 Working memory delayed-recognition task with affective distracters

Working memory was assessed as part of the computerized testing battery using a delayed-recognition working memory task (WMDA; see Jha et al., 2020). Participants were instructed to remember faces that were presented during an initial encoding phase. Military-relevant affective distracters were presented during a delay interval between encoding and retrieval. Each trial began with the presentation of a memory array (S1) for 3,000 ms during an encoding phase. S1 contained either two memory items (high mnemonic load) or one memory item paired with a noise mask (low mnemonic load). A delay interval of 3,000 ms occurred after S1, followed by a test item (S2) that was presented for up to 2,500 ms. On 50% of trials, S2 was one of the memory items that appeared in S1 (match trials). On the other 50% of trials, S2 was a novel image that did not appear in S1 (non-match trials) or elsewhere in the experiment. Participants indicated whether S2 matched either memory item in S1.

Task-irrelevant distracters (either neutral or negative images) were presented for 2,000 ms during the delay interval, and images were preceded and followed by a fixation cross for 500 ms. Distracter images were selected from the Military Affective Picture System (MAPS; Goodman et al., 2016) image set. Negative and neutral image categories differed in their valence and arousal based on normative ratings reported previously by Goodman et al. (2016). Negative images occurred on half of trials and neutral images occurred on the other half. Participants completed a practice block of 16 trials (with feedback provided about their accuracy), followed by three experimental blocks consisting of 32 trials each (for a total of 96 trials).

Task demands were therefore manipulated along two levels of mnemonic load (low vs. high) and two levels of distracter’s valence (neutral vs. negative). This resulted in four distinct trial types: low load-neutral distracter, low load-negative distracter, high load-neutral distracter, and high load-negative distracter. Trial types occurred with equal frequency. Overall WMDA accuracy (% trials correct from experimental blocks) was calculated for each individual collapsing across all trial types. In Study 1, 93 participants in total completed all three blocks of the WMDA. Three additional participants were subsequently removed from all analyses because they responded to less than two thirds (<66%) of all trials. In Study 2, 231 participants in total completed all three blocks of the WMDA, but 2 additional participants were removed because they responded to less than two thirds (<66%) of all trials.

### 2.4 Operationally relevant drills in an augmented reality simulation environment

The Conflict Kinetics “Gunfighter Gym”^2^ is an augmented reality combat simulation system used for military training and evaluating individual and small-team operational and combat arms performance. The Gun Fighter Gym was installed and operated at each participating U. S. Army installation by the Conflict Kinetics staff. Participants first completed a series of baseline drills assessing combat arms performance and marksmanship to ensure basic soldier competencies and familiarize participants with the simulation procedures before proceeding to the two operationally relevant cognitive drills. These drills are described in more detail in Supplementary material. The entire squad of participants completed each drill before the squad moved on to the next. Each participant was assigned to utilize one of 12 firing lanes for the duration of the assessment session.

Participants used a synthetic M4 rifle to interact with elements of the augmented reality environment projected into the environment by video projectors. The synthetic rifles are laser-equipped to interface with video projectors and sensors to collect precision information about marksmanship accuracy and response time. The system utilizes accurate ballistics. Elements of the augmented reality environment are updated in real time in response to feedback from the rifle. The synthetic M4 rifles are indistinguishable from actual rifles: rifles must be fully “reloaded” with new magazines when empty, they have the same weight and feel and provide haptic feedback to the user when fired using compressed air. Unlike other simulation systems widely employed by the U. S. Army for training and evaluation (i.e., Engagement Skills Trainer, EST2000),^3^ the Conflict Kinetics system and synthetic rifles allow a full range of dynamic movement within the environment because they are not tethered to a pneumatic line. Participants completed several sets of drills described below: the first sets involved baseline drills ensuring familiarization with the system and assessment of basic marksmanship before moving to the third set of drills examining more complex decision making. Drills were restarted when technical errors interrupted the proper completion of a drill, and measures of performance were obtained from the complete, uninterrupted drill.

In Study 1, 120 total participants attended the operational performance assessment. Two participants were excluded from analyses of all operational measures because they arrived with an arm injury that affected their use of the synthetic rifle or did not bring corrective lens, respectively. In Study 2, 223 total participants attended the operational performance assessment.

#### 2.4.1 Shoot/Do Not Shoot drill

Participants completed a Shoot/Do Not Shoot drill that assessed their ability to make speeded and accurate decisions about the threat of a potential target or non-target from the standing ready position. Participants were instructed to engage threatening stimuli (Shoot trials) as quickly as possible, while refraining from engaging non-threatening stimuli. (Do Not Shoot trials). There were 25 Shoot trials and 5 Do Not Shoot trials in total. Threatening “Shoot” stimuli could be readily identified as life-size static images of hostile individuals holding a weapon pointed toward the firer, whereas non-threatening Do Not Shoot stimuli were presented as static images of individuals with no weapon. Stimuli were presented for only 1,000 ms, requiring participants to fire their weapon quickly before Shoot trials ended.

The primary outcome of interest was *Do Not Shoot* accuracy, defined as the percentage of Do Not Shoot trials in which participants correctly refrained from firing their weapon. To provide a comprehensive account of performance on this drill, shoot accuracy was also calculated as the percentage of Shoot trials in which participants correctly engaged the target within the 1,000 ms stimulus presentation window. Additionally, response time was measured for Shoot trials in which participants successfully engaged the target. These secondary outcomes are reported in Supplementary material.

In Study 1, data was lost from one participant during collection due to an error, one participant was excluded from analyses of these dependent measures because they fired less than eight total shots during the task, and three participants were excluded from analyses because they had exceptionally low accuracy on Shoot trials (<10.9% accuracy, 3 *SD* below the mean). In Study 2, two participants were excluded from analyses because they fired less than eight total shots during the task. Three participants were further excluded because of low accuracy on Shoot trials (<9.6% accuracy, 3 *SD* below the mean).

#### 2.4.2 Working memory (WM) Shoot Drill

Participants completed a WM Shoot Drill that assessed their ability to remember target stimuli over a short retention period before shooting the target stimuli in the presence of several distracters. At the beginning of each trial, participants were shown two to-be-remembered stimuli: a face stimulus (Target 1) and an object stimulus (Target 2). Then, the display was replaced with eight stimuli: the two targets along with six distractors. All eight stimuli (the two targets and six distractors) moved slowly in random directions along a straight line. Given the goal of the task to assess working memory, participants were instructed to remember the two target stimuli in a correct order while ignoring the distractors. Participants were required to engage the targets in the correct order: first Target 1, then Target 2. Once the correct target was successfully hit and removed, it disappeared from view. Sometimes, more than one hit was needed to remove a target (*M* = 1.17 hits per target, *SD* = 0.011) if the hit was too far from the mass center of the target. The trial ended once both target stimuli were successfully engaged. There were 15 trials in total, with a total of 30 targets. WM Shoot accuracy was measured as the percentage of shots that hit the correct target in the correct order. Any shot that hit a distractor or Target 2 while Target 1 was still visible was counted as an error.

### 2.5 Performance motivation and assessment feedback

Participants in Study 1 answered a series of six questions at the end of the laboratory -based assessment session about their prior experience in the Conflict Kinetics Gunfighter Gym. Questions investigated participants’ performance motivation during the simulation tasks and understanding of task instructions. Participants were asked to “express their own views about the marksmanship drills and scenarios they completed in the augmented reality simulation environment” by rating 4 statements relating to their motivation to perform well, and 1 statement assessing their understanding of the task instructions, using a scale from 1 (“not at all”) to 7 (“very much”). The statements were: (1) “I was committed to my performance goals during the marksmanship drills and scenarios,” (2) “I was motivated to perform well during the marksmanship drills and scenarios,” (3) “I was determined to succeed on the marksmanship drills and scenarios,” (4) “I gave the drills and scenarios my full attention,” and (5) “I understood the instructions during the marksmanship drills and scenarios.” Finally, participants provided any other written comments they had about the marksmanship drills and scenarios. Ninety-six participants completed the simulation assessment by the time they provided feedback during the laboratory-based assessment about their experiences during the simulation assessment. Participants in Study 2 completed the laboratory-based assessment before their attendance at the simulation assessment. As such, their feedback about the simulation session was not collected for this assessment.

### 2.6 Analysis

Our primary analyses utilized bivariate Pearson correlations to examine the relationships between laboratory-based tasks and their corresponding operationally relevant drills. Specifically, we tested associations between SART *A′* and Do Not Shoot accuracy, as well as between WMDA accuracy and WM Shoot Drill accuracy. These associations were central to the study, as each laboratory task and its corresponding drill share a high degree of overlap in attentional control demands, such as selecting and maintaining relevant information while suppressing inappropriate responses and inhibiting distractions. Given that Do Not Shoot accuracy scores may not be normally distributed because scores were limited to a small set of discrete values resulting from few total Do Not Shoot trials, we also verified the significance of these associations using rank-order Spearman correlations. Additionally, analyses controlling for basic marksmanship scores (i.e., scores on the Table V marksmanship assessment) were conducted and are reported in Supplementary material.

In addition, to assess consistency in results between the two studies, we evaluated the significance of the pooled correlations across studies using random effects meta-analysis. Meta-analytic effects were estimated using restricted maximum likelihood and weighted based on the inverse variance using the *R* package *metafor* (Viechtbauer, 2010).

## 3 Results

Descriptive statistics are reported in Table 1 describing dependent measures calculated from the two laboratory-based cognitive tasks and two simulation drills. The descriptive statistics for all outcomes are presented in Supplementary material.

**Table 1:** Descriptive statistics of cognitive and operational performance measures.

Measure	Study 1		Study 2	
	*N*	Mean (*SD*)	*N*	Mean (*SD*)
Cognitive tasks				
SART *A′*	99	0.789 (0.106)	216	0.878 (0.080)
WMDA accuracy	90	71.368 (10.477)	229	74.840 (9.226)
Operationally relevant drills				
Do Not Shoot accuracy	113	70.973 (20.527)	218	77.890 (18.743)
% Shots correct	118	54.932 (15.897)	223	67.167 (16.041)

Means and standard deviations (SD) are provided for dependent measures of cognitive and operational performance in Study 1 and Study 2. The sample size for each measure is provided following exclusions.

### 3.1 Cognitive correlates of simulated operational performance

#### 3.1.1 Study 1

**Figure 1:**
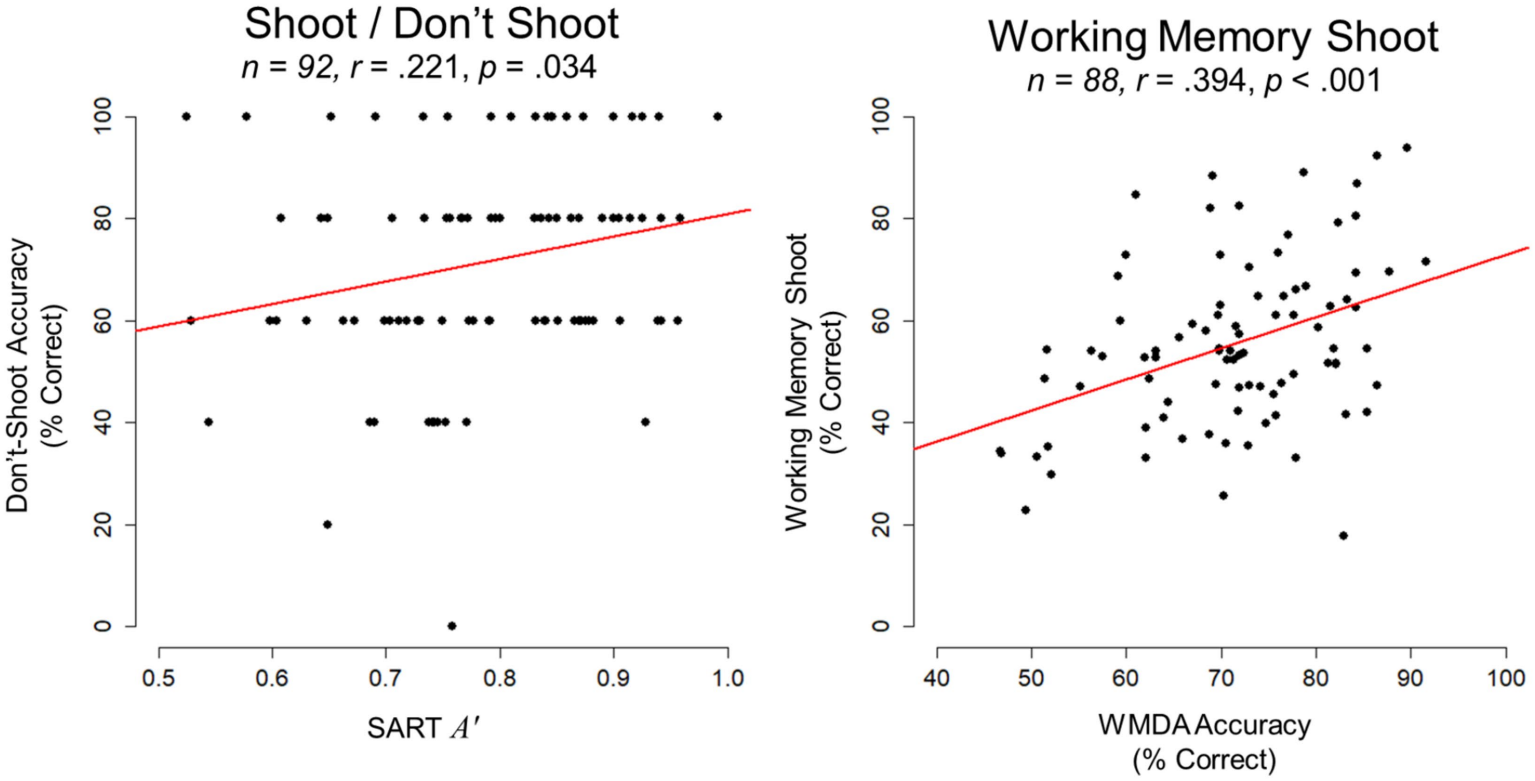
Scatterplots for Study 1 depict the association between Do Not Shoot accuracy in the Shoot/Do Not Shoot Drill and SART *A′* on the left, and the association between WM Shoot Drill accuracy (% shots correct) and WMDA accuracy on the right. Sample size and bivariate correlation coefficients (*r*) are provided.

SART *A′* was significantly correlated with Do Not Shoot accuracy (*r* = 0.221, *p* = 0.034, 95% CI [0.017, 0.408]) and this association was also significant when evaluated with rank-order Spearman correlations (*ρ* = 0.245, *p* = 0.019). WMDA accuracy was correlated with WM Shoot Drill accuracy (*r* = 0.394, *p* < 0.001, 95% CI [0.201, 0.558]). A summary of correlations are provided in Table 2 and scatterplots depicting these associations are shown in Figure 1.

**Table 2:** Correlations between cognitive and operational performance measures in Study 1 and Study 2.

	1	2	3	4
Study 1				
Cognitive tasks				
1. SART *A′*		82	92	97
2. WMDA accuracy	0.296**		83	88
Operationally relevant drills				
3. Do not-Shoot accuracy	0.221*	0.2		113
4. WM Shoot Drill accuracy	0.235*	0.394***	0.318***	
Study 2				
Cognitive tasks				
1. SART *A′*		216	200	208
2. WMDA accuracy	0.367***		211	220
Operationally relevant drills				
3. Do not-Shoot accuracy	0.165*	0.024		212
4. WM Shoot Drill accuracy	0.184**	0.038	0.118	

Bivariate correlations for Study 1 and Study 2 between cognitive and operational performance measures are provided in the lower diagonal of the table. The pairwise sample size for each correlation is provided in the upper diagonal. *p < 0.05, **p < 0.01, ***p < 0.001.

#### 3.1.2 Study 2

**Figure 2:**
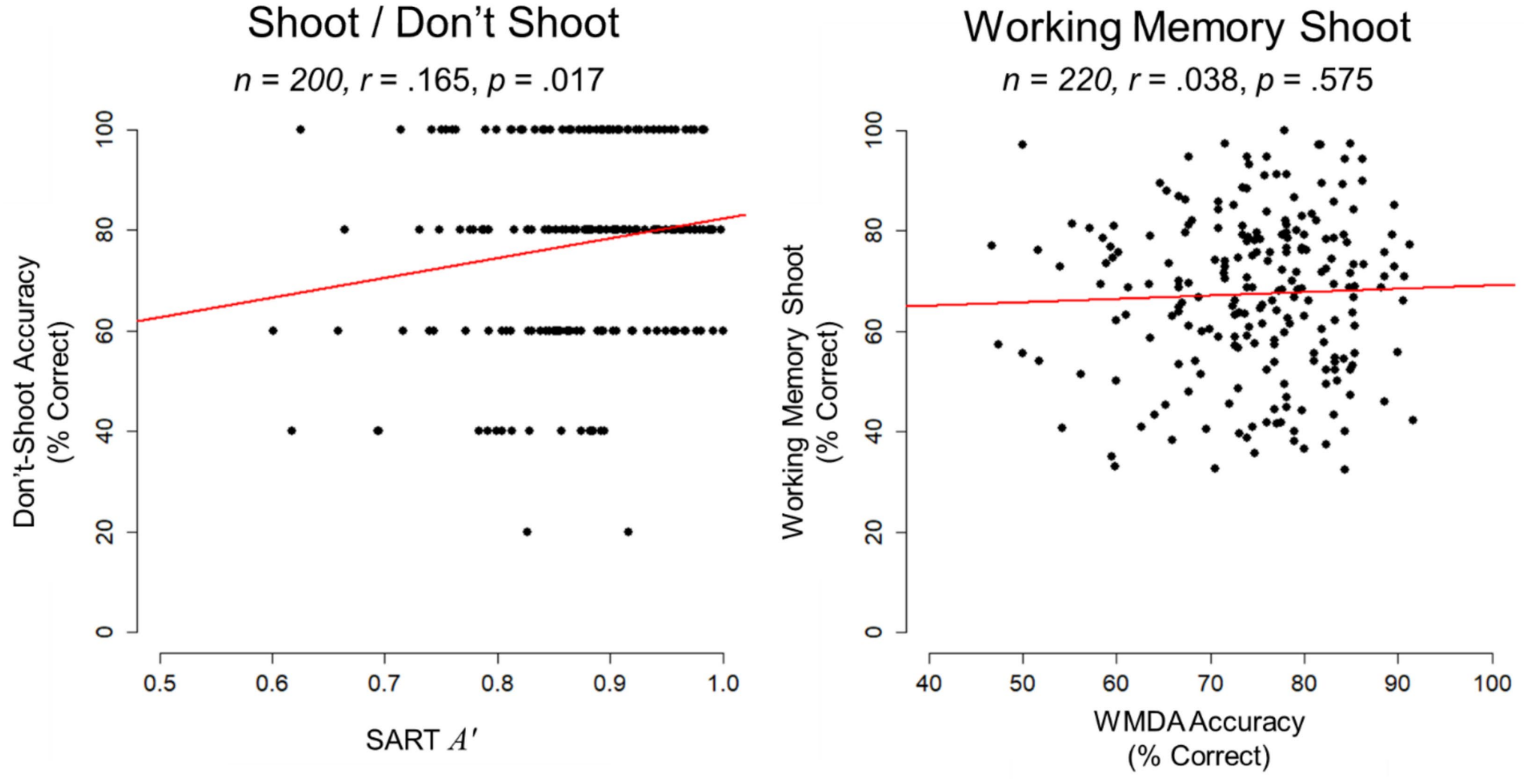
Scatterplots for Study 2 depict the association between Do Not Shoot accuracy in the Shoot/Do Not Shoot Drill and SART *A′* on the left, and the association between WM Shoot Drill accuracy (% shots correct) and WMDA accuracy on the right. Sample size and bivariate correlation coefficients (*r*) are provided.

SART *A′* was significantly correlated with Do Not Shoot accuracy (*r* = 0.165, *p* = 0.020, 95% CI [0.026, 0.297]). However, when evaluated with rank-order Spearman correlations, the association (*ρ* = 0.130, *p* = 0.067) did not reach significance. WMDA accuracy was not correlated with WM Shoot Drill accuracy (*r* = 0.038, *p* = 0.575, 95% CI [−0.095, 0.169]). A summary of correlations are provided in Table 2 and scatterplots depicting these associations are shown in Figure 2.

#### 3.1.3 Meta-analysis of pooled effects

We next aggregated these significant results across studies using random-effects meta-analysis. SART *A′* was significantly correlated with Do Not Shoot accuracy (*r* = 0.184, *p* = 0.001, 95% CI [0.073, 0.295]) based on the results of the meta-analysis. Meta-analysis of Spearman rank-order correlations was also significant for associations between SART *A′* and Do Not Shoot accuracy (*r* = 0.168, *p* = 0.003, 95% CI [0.057, 0.280]). Finally, WMDA accuracy and WM Shoot Drill accuracy (*r* = 0.212, *p* = 0.236, 95% CI [−0.139, 0.563]) were not significantly correlated.

### 3.2 Motivation and assessment feedback

To evaluate the simulation system as a high-fidelity training and testing environment, participants were asked about their experiences engaging in the simulation tasks. Participants were highly motivated to perform and succeed during the operational performance tasks based on their responses to questions about their experiences (see Table 3). Furthermore, participants reported that on average they understood the instructions (*M* rating = 6.469, *SD* = 1.248; rated from 1 to 7). These ratings suggest that errors in performance on the simulation drills were not due to poor understanding of the task instructions or low motivation to perform. Furthermore, the free-response feedback gathered from the participants supports the fidelity of the simulation system, as participants acknowledged the simulation tasks as a good opportunity for realistic training. For example, one participant remarked, “It was a really good training experience that we do not get. [I] personally think the army should incorporate [the simulation drills] into our training SOP.” Other participants referred to the drills in similar terms: “[The] marksmanship training was very useful as it pertains to my job and gave myself and my soldiers good effective training,” and “…the marksmanship drills gave myself and others the chance to learn and put our training to action.” All other free response comments (when provided) were likewise positive about the drills.

**Table 3:** Descriptive statistics of motivation to complete the operationally relevant drills.

Measure	Mean (*SD*)
I was committed to performance goals	6.344 (1.272)
I was motivated to perform well	6.375 (1.324)
I was determined to succeed	6.375 (1.292)
I gave the drills my full attention	6.135 (1.343)
I understood the instructions	6.469 (1.248)

Means and standard deviations (SD) are provided for individuals’ (*n* = 96) ratings of self-reported performance motivation during the simulation tasks and scenarios. Ratings (from 1 “not at all” to 7 “very much”) were collected during the online laboratory-based assessment following completion of the simulation tasks.

## 4 Discussion

The present studies examined associations between cognitive abilities, indexed by performance on laboratory-based tasks of attentional control, and performance on simulated operationally relevant drills completed by active-duty U.S. Army infantry soldiers. Specifically, participants completed laboratory-based cognitive assessments of sustained attention and working memory along with drills requiring attentional control in an augmented reality simulation environment. We found that cognitive task performance was positively correlated with simulated operationally relevant analogs. These findings highlight the importance of individual differences in attentional control for complex and speeded decision making in the military operational context and support the use of laboratory-based tasks for measuring core cognitive processes relevant to real-world military operational contexts.

Attentional lapses and errors of commission are common in laboratory tasks used to assess attentional control and response inhibition. Our findings suggest that these lapses are also frequent in more ecologically valid situations: individuals made errors of commission in the operationally relevant analog of a continuous performance response inhibition task—the Shoot/Do Not Shoot Drill—roughly 20–30% of the time. Importantly, individuals’ tendency to make errors of commission (i.e., Do Not Shoot errors) in the Shoot/Do Not Shoot Drill was associated with their SART performance accuracy (indexed by *A’*). This association, though modest (Study 1 *r* = 0.221, *n* = 92; Study 2 *r* = 0.165, *n* = 200), was significant, and consistent across both studies as demonstrated by the meta-analysis of results from both studies. Moreover, the magnitude of the correlation is consistent with prior research linking laboratory-based cognitive performance to operationally relevant outcomes (Biggs et al., 2015; Biggs and Pettijohn, 2021; Brunye et al., 2024). Taken together, these findings suggest that laboratory-based cognitive task performance may be a reasonable predictor of operationally relevant performance with potential to influence high-stakes situations such as live-fire engagements. However, as noted by Brunye et al. (2024), further interdisciplinary research is needed to fully identify all relevant predictors of operational performance.

We found less reliable associations between performance on a delayed-recognition working memory task performance and the simulated working memory drill requiring participants to correctly recall and engage to-be-remembered stimuli with their rifle. Indeed, working memory is thought to be critically involved in the execution of cognitively complex tasks, and this ought to be true of tactical scenarios in which individuals must quickly identify and select goal-relevant targets for engagement in line with operational objectives. Nevertheless, performance on the Working Memory Delayed-Recognition (WMDA) task was only significantly associated with performance on the Working Memory Shoot Drill in Study 1, and the pooled effect across studies was not significant. As such, future research is needed to confirm the magnitude and significance of associations between laboratory-based measures of working memory and those assessed in simulated operational tasks.

Self-report feedback from participants in this study supports the use of augmented reality simulation tasks. Soldiers reported high levels of focus and determination to succeed, and their free-response feedback emphasized the value of realistic training and assessment through the simulation system. There was no evidence that performance on the simulated drills was affected by misunderstandings of the tasks or lack of motivation. However, we did not explicitly ask participants about the ecological validity of the simulations; therefore, our conclusions are inferred from their self-reported ratings and free-response feedback regarding their experiences.

The use of virtual and augmented reality simulation is increasingly recognized as an essential component for training by the U.S. military. Virtual and augmented reality simulations are already incorporated into training and assessment procedures for infantry soldiers in the U.S. Army, including their use in the training and assessment of rifle marksmanship and weapon proficiency (i.e., Engagement Skills Trainer; Department of the Army, 2019). The degree to which these simulations accurately assess abilities necessary for successful operational performance in the field is therefore important for their continued use and dissemination. As such, future studies should directly compare performance in simulated scenarios to standard “live-fire” assessments of marksmanship. Limited empirical evidence supports the validity of simulations used for this purpose. For example, prior research has found that marksmanship in simulated scenarios is strongly correlated with live-fire performance (Hagman, 1998; Schloo and Mittal, 2021).

Nevertheless, our findings support the view that performance on cognitive tasks assessing attentional control—specifically, the ability to select and maintain goal-relevant information while inhibiting irrelevant responses or distraction—may serve as meaningful predictors of operationally relevant performance in more naturalistic settings. This is theoretically significant, as attentional control is a core cognitive ability that underpins effective task execution, that could be particularly relevant for high-stakes environments like military operations. Yet, military assessments have traditionally prioritized aptitude evaluations, such as the Armed Services Vocational Aptitude Battery (ASVAB), which primarily measures crystallized intelligence while overlooking fluid intelligence, memory, and learning ability (Roberts et al., 2000). Researchers have suggested that the predictive validity of the ASVAB can be improved by accounting for individuals’ ability to control their attention (Martin et al., 2020), or that assessing other cognitive capacities, such as attentional vigilance, may be useful in predicting on-the-job performance in military contexts (Barron and Rose, 2017; Matthews et al., 2013; Trent and Barron, 2021).

Aside from their consideration as part of aptitude assessment and selection processes, attentional control and other cognitive abilities might also serve as direct targets for training interventions. For example, there has been growing interest in the use of computer-based cognitive training methods for dissemination within military populations (Blacker et al., 2019). Some initial research has suggested that training with a computer-based response-inhibition task can reduce commission errors during simulated shooting (Biggs et al., 2015). Alternatively, studies of mindfulness training with active-duty soldiers have shown that training can protect and bolster cognitive abilities such as attention and working memory, measured using the same laboratory-based tasks as in the present study (Jha et al., 2020, 2021; Zanesco et al., 2019). Associations between performance on cognitive tasks and performance on more ecologically valid analogs, as we observed in the present study, suggest that training-related benefits may mediate changes in operationally relevant performance. Some initial evidence supports this supposition, as mindfulness practice has been shown to be associated with improvements on marksmanship under physical stress (Nassif et al., 2023). Reductions in attentional lapses, commission errors, and failures in working memory could be consequential if they generalize to successful operational performance in the field, for military service members as well as civilian police officers. Future studies should continue to examine these questions by investigating whether the effects of mindfulness training and other cognitive training modalities on operational performance are mediated by their benefits in attention and working memory, as assessed on laboratory-based tasks.

## 5 Conclusion

The present study supports the use of cognitive tasks to assess soldiers’ attentional control, a domain-general capacity that is linked to operationally relevant performance in ecologically valid analogs. By demonstrating significant associations between cognitive performance in laboratory settings and simulated operational drills, our research contributes to understanding of the cognitive mechanisms underlying effective task execution in military contexts. As noted by others (Beal, 2010; Picano et al., 2017; Martin et al., 2020), incorporating attentional control assessments into soldier evaluations could offer valuable insights to inform selection for service and assignment to military occupational specialties.

## Data Availability

The original contributions presented in the study are included in the article Supplementary material, further inquiries can be directed to the corresponding author.

## Footnotes

^1^ https://www.millisecond.com/

^2^ https://www.conflictkinetics.com/

^3^ www.cubic.com
