# Supplementary material for: Examining cognition in action: laboratory-based attentional control tasks predict performance on combat-relevant tasks in an augmented reality training environment
Source: Front Psychol. 2025 May 5;16:1543161. doi: 10.3389/fpsyg.2025.1543161 (PMC12087572; doi:10.3389/fpsyg.2025.1543161)
Supplement: Supplementary file 1 [file Table_1.docx]

**Supplementary Material**

1. **Baseline Combat Arms Performance and Marksmanship Drills**

Four drills were completed in succession assessing M4 rifle marksmanship. The first three drills also served to familiarize participants with the synthetic rifle and the simulation system prior to completing the Table V M4 weapon qualification as a formal assessment of their marksmanship ability (Department of the Army, 2019).

***Standing, kneeling, and prone at 7 meters, 50 meters, and 100 meters (SKP 7M, 50M, and 100M).*** In the first drill, participants fired at 9 E-Type targets while moving between unsupported standing, unsupported kneeling, and unsupported prone positions. Participants were instructed to fire at the target 7 meters away while standing, fire at the 50-meter target while kneeling, and the 100-meter target while laying prone. There were three total targets per condition. Targets were presented one at a time, and participants fired until they hit each target. The next target appeared once the target was hit following a short delay. Trials were intermixed, requiring individuals to move fluidly between standing, kneeling, and prone positions while firing at different targets. Accuracy during the drill was calculated as the percentage of total shots fired that hit targets. In Study 2, accuracy was unavailable for six participants (members of the same squad).

***Standing and kneeling at 7 meters and 50 meters (SK 7M and 50M).*** In the second drill, participants fired at 9 E-Type targets while moving between an unsupported standing position and an unsupported kneeling position. Participants fired at the 7-meter target while standing and the 50-meter target while kneeling. There were five total targets in the 7-meter standing condition and four total targets in the 50-meter kneeling condition. Participants fired at each target one at a time until they hit the target, and accuracy was calculated as the percentage of total shots fired that hit targets.

***Standing and prone at 7 meters and 100 meters (SP 7M and 100M).*** In the third drill, participants fired at 9 E-Type targets while moving between an unsupported standing position and an unsupported prone position. Participants fired at the 7-meter target while standing and the 100-meter target while prone. There were five total targets in the 7-meter standing condition and four total targets in the 100-meter prone condition. Participants fired at each target one at a time until they hit the target, and accuracy was calculated as the percentage of total shots fired that hit targets.

***Table V M4 weapon qualification.*** Finally, participants completed the “Table V” M4 weapons qualification. For a U.S. Army Infantry Soldier to qualify as proficient with their rifle, they must complete a series of qualification courses that culminate in a live-fire timed assessment of rifle proficiency (i.e., Tables I through Table VI; Department of the Army, 2019). Table V is likewise a timed assessment of marksmanship and rifle proficiency that takes place in four phases and is completed as practice for the Table VI qualification. Table V requires participants to shoot at arrays of single and multiple stationary targets between 50-meters and 300-meters from four firing positions: the prone unsupported, prone supported, kneeling supported, and standing supported positions. Table V is identical to the Table VI weapons qualification in terms of the number and arrangement of presented targets, ordering of firing positions, and target distances. Table V, however, is purposely more challenging than the actual Table VI qualification event because targets are presented at a faster tempo.

Forty E and F-Type targets are presented in total during Table V, and participants are instructed to hit as many targets as possible in the limited amount of time each is presented. There were 10 total targets presented in each of four successive phases. Participants had three seconds to score a hit when an array of only a single target was presented, whereas they had five seconds for an array of two targets, 12 seconds for an array of three targets, and 16 seconds for an array of four targets. Participants are provided four magazines worth of ammunition in total to complete the four phases of the drill. The precise trial ordering and timing of target arrays is identical to that used in the actual course of fire (see E-38; Department of the Army, 2019). The total number of targets hit were calculated as participants’ Table V score. The firer must hit more than 23 total targets (out of 40) in the allotted time to receive a qualifying score. In Study 2, Table V scores were unavailable for two participants.

**Combat Response Time**

Response time was assessed through speeded performance on two drills in which participants fired at targets from a ready position (i.e., the weapon is aligned straight ahead towards the field in which the target is expected to appear) and a low-ready position (i.e., the weapon is relaxed and oriented down and must be lifted to fire at the target when it appears). The finger is held off the trigger in the low-ready position. Participants are instructed to hit the target as quickly as possible in both positions when it appears. Participants first completed seven successive trials in the ready position before completing seven successive trials in the low-ready position with a short break between ready and low-ready drills. Targets were present until they were hit, but response time was calculated only from trials in which participants hit the target with their first shot. Three participants were excluded from calculation of low-ready response times because they had fewer than four accurate trials available (i.e., < 57% accuracy). In Study 1, participants hit the target on the first try 99.2% (*SD* = 3.9%) on average in the ready position and 87.8% (*SD* = 13.8%) on average in the low-ready position. In Study 2, participants hit the target on the first try 98.2% (*SD* = 5.2%) on average in the ready position and 89.4% (*SD* = 12.9%) on average in the low-ready position.

**Supplementary Results**

An examination of characteristics of combat arms performance indicated that participants were generally proficient with the M4 rifle and understood task instructions (see Supplementary Table 1). Performance was high in simple marksmanship tasks (e.g., SKP 7M 50M 100M) but worse in those requiring more complex, speeded decision making. There are a few noteworthy aspects of performance to highlight. In Study 1, the Table V qualification score was acceptable on average (*M* = 22.58, 95% CI [21.3 to 23.8]), and the 95% CI overlapped with a qualifying score of 23. 52.5% of participants achieved a qualifying Table V score. In Study 2, however, the Table V qualification scores were below qualifying levels on average (*M* = 18.76, 95% CI [17.9 to 19.6]). Together, performance on these drills confirms individuals’ marksmanship proficiency and the potential need to account for between-person differences in marksmanship when examining associations between cognitive task performance and simulated drills.

**Study 1.** Associations between SART and WMDA performance and Don’t-Shoot accuracy and WM Shoot Drill accuracy, respectively, remained significant after controlling Table V marksmanship scores in a multiple regression model, and neither SART *A′* (*r* = .054, *p* = .597, 95% CI [-0.147, 0.251]) nor WMDA accuracy (*r* = .041, *p* = .707, 95% CI [-0.170, 0.248]) were significantly correlated with Table V marksmanship scores.

**Study 2.** Associations remained significant after controlling marksmanship scores for marksmanship scores in a multiple regression model, and neither SART *A′* (*r* = .027, *p* = .699, 95% CI [-0.110, 0.163]) nor WMDA accuracy (*r* = .013, *p* = .853, 95% CI [-0.120, 0.145]) were significantly correlated with Table V marksmanship scores.

| Supplemental Table 1: Descriptive Statistics of all Operational Performance Measures | | | | |
| --- | --- | --- | --- | --- |
|  | Study 1 | | Study 2 | |
| Measure | *N* | Mean (*SD*) | *N* | Mean (*SD*) |
| Combat Arms Performance |  |  |  |  |
| SK 7M 50M | 118 | 92.066 (9.037) | 223 | 83.166 (12.797) |
| SP 7M 100M | 118 | 90.897 (11.351) | 223 | 78.042 (19.357) |
| SKP 7M 50M 100M | 118 | 90.714 (12.240) | 217 | 76.844 (18.876) |
| Table V Score | 118 | 22.576 (6.877) | 221 | 18.764 (6.443) |
| Combat Response Time |  |  |  |  |
| Ready RT (msec) | 118 | 696.550 (570.763) | 223 | 788.532 (627.502) |
| Low-Ready RT (msec) | 115 | 1559.530 (1027.361) | 223 | 1913.233 (1015.217 |
| Shoot / Don’t-Shoot |  |  |  |  |
| Shoot Accuracy | 113 | 77.522 (18.634) | 218 | 71.064 (18.822) |
| Shoot RT (msec) | 113 | 654.556 (58.424) | 218 | 662.986 (68.784) |
|  |  |  |  |  |

Note: Means and standard deviations (*SD*) are provided for dependent measures of cognitive and operational performance in Study 1 and Study 2. The sample size for each measure is provided following exclusions. MW = mind wandering; RT = response time; SKP = standing, kneeling, prone % accuracy; WMDA = Working Memory Delayed-Recognition task
